# What Makes Medical Education Meaningful: A Qualitative Evaluation of Student and Educator Perspectives on UK Clinical Education

**DOI:** 10.7759/cureus.107280

**Published:** 2026-04-18

**Authors:** Alex Kurowski-Ford, Patrick Sharman, Jessica Savill, Iqra Shamim, Pooja Siddhi

**Affiliations:** 1 Medical Education, Walsall Healthcare NHS Trust, Walsall, GBR; 2 Social Sciences, University of Sheffield, Sheffield, GBR; 3 Medical Education, University Hospitals of North Midlands NHS Trust, Stoke-on-Trent, GBR; 4 Neonatology, Walsall Healthcare NHS Trust, Walsall, GBR

**Keywords:** blended learning, clinical education, clinical placements, curriculum alignment, feedback in medical education, qualitative study, student engagement, thematic analysis, undergraduate medical education

## Abstract

Background

Undergraduate clinical education has evolved to encompass a broad integration of online, in-person, simulation-based, and bedside teaching modalities. Although these formats are now embedded in post-pandemic curricula, concerns persist regarding student engagement, preparedness for clinical practice, and the capacity of educators to deliver high-quality, relational teaching within pressured clinical environments. Understanding both student and educator perspectives is essential for informing effective curriculum design.

Methods

Semi-structured interviews were conducted among 6 UK medical students and 11 medical educators. Data were analyzed using an inductive approach to thematic analysis following Braun and Clarke’s six-phase framework. The student and educator datasets were analyzed separately before thematic integration.

Results

Five overarching themes were identified. First, participants emphasized the value of a blended teaching model, noting the complementary strengths of simulation for confidence-building and bedside teaching for authenticity and professional identity formation. Second, interactive, case-based, and relational pedagogy was favored over didactic lectures, with continuity of teaching staff and opportunities for active participation seen as central to engagement. Third, feedback was regarded as most effective when it was timely, specific, and dialogic, although its delivery appeared to be constrained by service pressures. Fourth, readiness and motivation to learn were shaped by the level of structure and pastoral support available on placements, with students reporting difficulties navigating self-directed learning. Finally, misalignment between curricula and assessment was viewed as a major barrier to meaningful clinical engagement.

Conclusion

Students and educators shared preferences for a structured, practice-aligned educational approach incorporating blended teaching modalities, interactive pedagogy, high-quality feedback, and clear alignment between curriculum and assessment. Addressing curriculum overload, clarifying expectations, and recognizing the pastoral contributions of educators were identified as important considerations for institutions. These findings provide insight into the factors influencing student engagement and educator effectiveness in the evolving post-pandemic pedagogical landscape.

## Introduction

Clinical placements remain the cornerstone of undergraduate medical education in the UK. They are the arena in which students develop knowledge, skills, and professional behaviors within authentic clinical environments. Regulatory bodies require medical graduates to demonstrate expert communication, clinical reasoning, and procedural skills, necessitating rigorous evaluation of the teaching modalities and approaches used to educate medical students [1].

To meet these standards, medical schools now employ a diverse spectrum of clinical teaching modalities, including in-person and online lectures, small-group seminars, high-fidelity simulation, and bedside teaching [2]. In the years following the acute phase of the COVID-19 pandemic, many emergency adaptations to clinical teaching have now been consolidated into stable curricular designs [3]. As a result, the landscape of medical education has evolved considerably, with online, hybrid, and simulation-based teaching playing increasingly central roles [4].

While early evidence suggests these changes have yielded important benefits, with students citing greater accessibility and flexibility of online teaching methods [5], concerns persist about student engagement, motivation, and preparedness for clinical practice. Students describe diminished confidence in patient-facing encounters and clinical skills [6], with corresponding decreases in perceived preparedness and opportunities for practical learning [7]. These challenges appear to extend beyond clinical competencies, with reports of waning student engagement, motivation, and attendance across several cohorts [8,9]. Similarly, medical educators report increasing difficulty in maintaining learner interest and in fostering active participation, both online and in clinical settings [10].

Although the type of evidence regarding the effectiveness of different teaching modalities is highly nuanced, recent declines in student engagement, motivation, and practical competence, combined with educator-reported challenges in cultivating participation, highlight the need to examine both student and educator perspectives on contemporary teaching approaches. Understanding these viewpoints is essential for informing approaches to student disengagement and supporting educator effectiveness in the evolving post-pandemic learning environment.

Aims

This study aims to explore student and educator perspectives on the usefulness, effectiveness, and acceptability of current teaching modalities and approaches within undergraduate clinical education settings. A secondary aim is to examine how these perspectives might inform teaching delivery and institutional structures to better support both learners and educators.

Through qualitative analysis of student and educator experiences relating to teaching modalities, assessment, feedback, engagement, and motivation, this study seeks to provide insight into the factors influencing the delivery and reception of contemporary undergraduate clinical education.

Related abstracts containing summary data from this article have been accepted for poster presentation at the 4th EURACT Medical Education Conference, April 23-25, 2026, and for presentation at the AMEE Conference, August 22-26, 2026.

## Materials and methods

Design

A qualitative methodology was considered most appropriate to fulfil the study’s aim of exploring perceptions, preferences, and learning experiences, rather than to compare objective educational outcomes. Semi-structured interviews were used to elicit in-depth accounts from participants, while allowing interviewers to explore emerging topics of interest through follow-up questioning. The interview guides were developed iteratively in line with the study aims, informed by areas of limited qualitative exploration in the existing literature. An inductive approach to inquiry was adopted to enable data-driven analysis of participants’ responses.

Participants

Seventeen participants were interviewed, comprising 6 UK medical students and 11 medical educators. Students were recruited following the completion of a multicenter electronic survey exploring attitudes towards teaching modalities and approaches, disseminated through university mailing lists, professional networks, and weekly bulletins. Inclusion criteria required participants to be current or recently graduated (within one year) medical students from a UK medical school. At the end of the survey, the respondents were invited to voluntarily register their interest in participating in an interview, from which a purposive sample was selected. The student interviewees included five clinical-year students (two third-year, one fourth-year, and two fifth-year students) and one foundation-year doctor, representing three UK medical schools.

Educators were recruited voluntarily through existing academic and professional networks. Eligible participants were required to have active roles in undergraduate medical education in the UK. Participants were purposively recruited to represent a range of professional backgrounds and levels of experience, including clinical teaching fellows, consultants, advanced care practitioners, operating department practitioners, and general practitioner tutors, based across the local National Health Service (NHS) Trust.

All participation was voluntary and non-incentivized. A purposive sampling strategy was used to capture a range of perspectives across different stages of training and teaching contexts. Recruitment continued until sufficient depth and diversity of perspectives were achieved.

Data collection

Interviews were conducted virtually via Microsoft Teams between April and July 2025. Participants were offered 60-minute appointments, with a mean interview duration of 52 minutes. Informed consent was obtained for audio recording and transcription. The interviews were transcribed using the live transcription feature available on Microsoft Teams. Transcripts were manually cross-checked against audio recordings to ensure accuracy, after which all audio files were deleted. Each transcript was reviewed independently by AK-F, IS, and PS to remove identifying information, and all data were anonymized prior to analysis.

**Figure 1:**
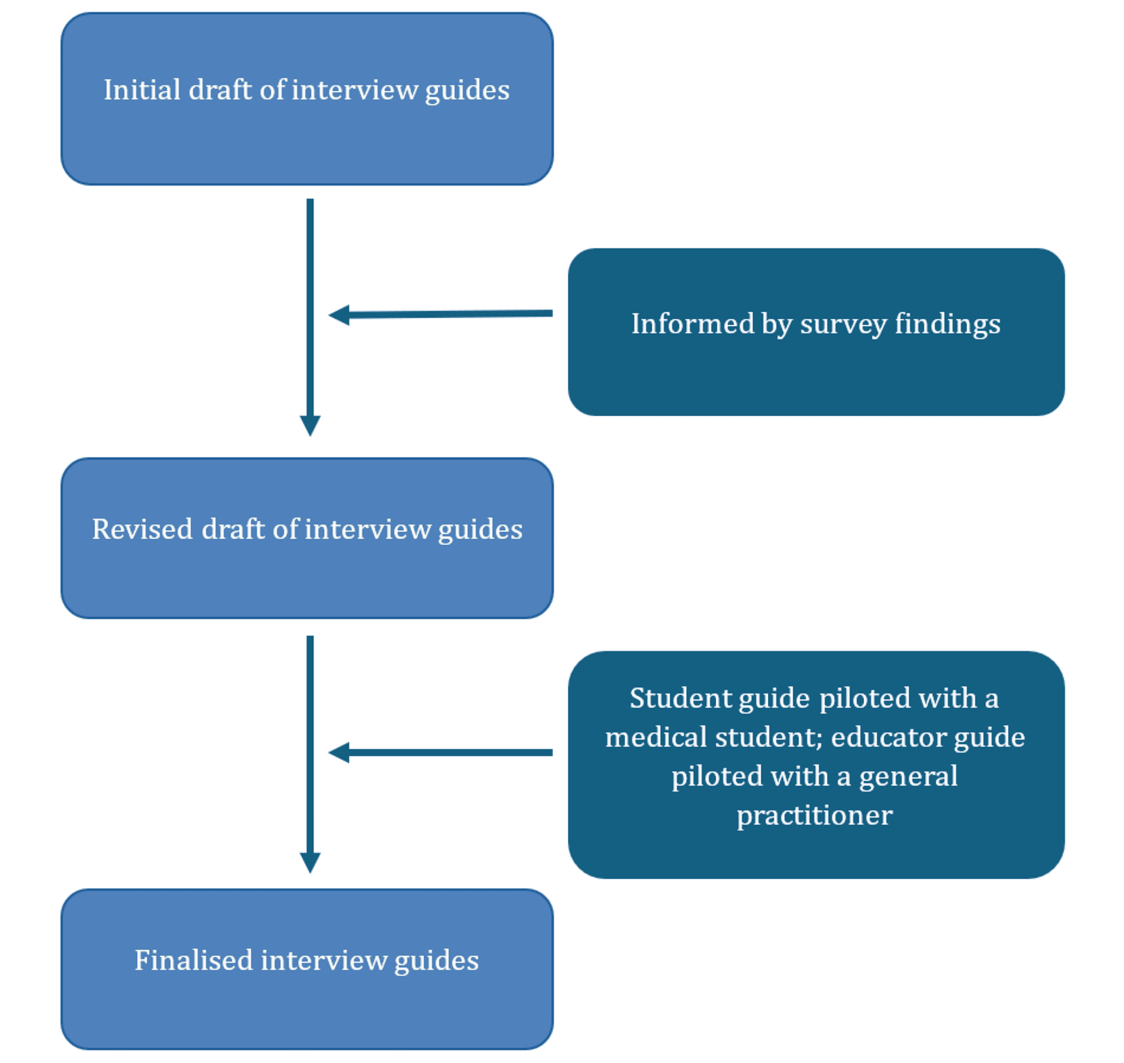
Flowchart demonstrating the process of semi-structured interview guide development Note: This figure was created by the authors using Microsoft Word (Version 2603; Microsoft Corp., Redmond, WA, USA). No generative AI tools or AI-assisted image-generation features were used.

Student interviews were conducted by AK-F. Educator interviews were conducted by AK-F and IS. Semi-structured interview guides were developed iteratively, informed by the study team’s experience, findings from the preceding survey, and pilot interviews (Figure 1). Separate but conceptually aligned guides were used for students and educators. The interview guides were piloted with one volunteer student and one educator, although no substantial amendments were required. Interview guides are provided in Appendices A and B. Field notes were taken during and immediately after the interviews to support contextual interpretation.

Data analysis

**Figure 2:**
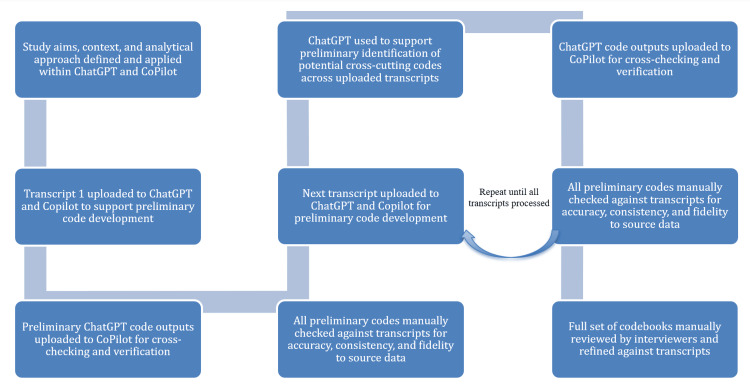
Flowchart demonstrating AI-assisted preliminary coding and codebook development Note: This figure was created by the authors using Microsoft Word (Version 2603; Microsoft Corp., Redmond, WA, USA). No generative AI tools or AI-assisted image-generation features were used.

Data analysis was informed by Braun and Clarke’s six-phase approach to thematic analysis [11]. This approach was selected to enable a data-driven interpretation of participant narratives, while retaining the flexibility to capture both shared and divergent perspectives across the dataset. Figure 2 illustrates the workflow of artificial intelligence (AI)-assisted preliminary coding and subsequent human-led codebook development used in this study. AI-assisted analysis was used to support systematic preliminary coding and the identification of potential cross-cutting concepts. ChatGPT and Microsoft Copilot were selected for this purpose, informed by emerging literature suggesting that large language models may assist preliminary qualitative coding when used with appropriate human oversight [12,13]. Transcripts were fully anonymized prior to analysis.

All AI-generated outputs were manually reviewed and refined through a structured, multi-step human verification process conducted by both interviewers (AK-F and IS). This involved checking all preliminary codes against the original transcripts to ensure accuracy, representativeness, and alignment with the source data, as well as incorporating additional codes and insights derived from independent review and reflexive observations. Codebooks were then developed iteratively through repeated engagement with the data, and earlier coded transcripts were revisited as subsequent interviews were analyzed to refine the codebooks and identify concepts that became more salient across the dataset. AI tools were used only to support preliminary coding and codebook development; final coding decisions, interpretation, and theme development were led by the study team. Where discrepancies occurred, human interpretation took precedence.

Following finalization of the codebooks, the codes were grouped into subthemes for student and educator datasets separately. These subthemes were then compared across groups to identify areas of convergence and divergence. Subthemes were subsequently synthesized into overarching themes through ongoing comparison across transcripts. The final themes were developed collaboratively among the study team through iterative discussion and consensus. Human-led reflexive engagement was maintained throughout the analysis process, incorporating interviewers’ observations, field notes, and contextual insights from the interviews.

Author positionality

The authors acknowledge that their roles as clinicians and medical educators may have influenced the design and interpretation of this study. Their experiences within UK medical education may have shaped the development of the interview questions and the interpretation of participant responses. To address this, the authors sought to remain reflexive throughout the study process, considering alternative perspectives and ensuring that themes reflected the breadth of views expressed by participants. The authors incorporated frequent reflection, discussion, and consideration of both their individual and collective experiences throughout all stages of the study.

Ethical considerations

This study involved interviews with educators and students regarding local educational methods and did not involve NHS patients or identifiable patient data. This project was reviewed by the hosting institution’s research governance team using the UK Health Research Authority (HRA) decision tool and classified as a service evaluation; therefore, a formal NHS Research Ethics Committee (REC) review was not required under UK national regulations. Verbal informed consent was obtained from all participants before interview commencement, including consent for audio recording and the use of de-identified, anonymized quotations in analysis and publication. Participation was voluntary, and withdrawal was permitted at any time. Transcripts were de-identified and anonymized, securely stored in restricted-access files, and handled in accordance with institutional policy and UK data protection regulations.

## Results

Thematic analysis of the 6 student and 11 educator interview transcripts identified five main themes relating to the study aims. As shown in Table 1, these themes were developed from subthemes arising across both participant groups through analysis of codes generated from all 17 transcripts. While some subthemes were more strongly represented in one participant group than the other, all five main themes were derived from the combined analysis of the full dataset.

**Table 1:** Themes and subthemes derived from thematic analysis of student (S) and educator (E) interviews

Theme	Subthemes	Contributing group(s)
1. A blended approach to teaching is most effective	Advantages and disadvantages of online learning	S + E
	Trade-offs between simulation-based and bedside teaching	S + E
	Complementary strengths and purposes of different teaching modalities	S + E
	Operational realities and barriers to teaching	E
2. Interactive, case-based, and relational pedagogy best engages students	Educator and institutional factors driving engagement	S + E
	Student agency and peer networks	S + E
	Pedagogy and session design	S + E
3. Effective feedback is timely, specific, dialogic, constructive, and actionable	Preferred feedback is direct, actionable, constructive, and dialogic	S
	Poor feedback is generic, inconsistent, or absent	S
	Preferred feedback is immediate, dialogic, and psychologically safe	E
	Relative lack of feedback for educators	E
4. Supporting readiness and motivation requires scaffolding and pastoral care	Uncertainty regarding student autonomy and self-directed learning	S
	Student readiness, confidence, and autonomy	S + E
	Pastoral care as a support for student confidence and engagement	S + E
	An unrecognized role of educators in pastoral support	E
5. Aligning curriculum and assessment with clinical practice enhances relevance and learning	Curriculum overload as a barrier to meaningful learning	S + E
	Perceived misalignment between curriculum, assessment, and practice	S + E
	Assessment structure and formats	S + E

Theme 1: A blended approach to teaching is most effective

Students and educators advocated for the blended use of online and in-person teaching modalities, favoring a hybrid approach over single-modality models. Students generally found online learning to be less engaging than in-person sessions, describing it as “tedious” (S1, S3), “disinteresting” (S2), “unstimulating” (S4), and “dull” (S5). Several students described difficulty concentrating without the accountability of face-to-face interaction:

“Many of my colleagues struggle to engage online. And just, you know, video cameras off, microphones off, so they wouldn't engage.” (S1)

Educators similarly reported that virtual settings reduced opportunities for personal connection and interaction, with the “level of feedback, the personal approach…I think that’s what’s lost” (E4). Despite these limitations, online learning was valued for its flexibility and accessibility, particularly by reducing barriers related to travel and cost. Student 2 also praised recorded material for its "rewind-ability" and easier note-taking: “I like that I can pause, and I can adjust the speed, and I can take my time doing notes.”

Students and educators alike also described clinical education as most effective when the specific modalities of simulation teaching and bedside teaching were used in combination. Students associated simulation with a safe and engaging learning environment where confidence can be built prior to entering real clinical settings: “It's a low-pressure environment… you can make any mistakes and it's not going to cause any damage” (S3). Educators echoed this sentiment, referencing the value of simulation as a confidence-builder for early clinical years, and also praised its capacity to deliver standardized exposure to high-yield presentations. In addition to this, they highlighted the benefits of allowing students to rehearse rare but critical clinical events, with one educator reflecting, “you can’t always get to see some things… So to be able to get that practice in, I think is brilliant” (E11). However, both groups highlighted limitations: some students described feeling uncomfortable or hesitant during simulated learning experiences, while educators raised concerns that low-fidelity simulations can feel artificial and educationally limited. As one explained, “with simulation it is as high fidelity as you want it to be. But it is not a real patient. And there are limits to that” (E10). Educators also emphasized the substantial resource and logistical demands of simulation, noting that “if we haven't organized the rooms, if we haven't organized the equipment, if we haven't organized everything, then you're not going to be able to do the simulation” (E11).

Bedside teaching was praised by both groups of interviewees for its high degree of authenticity and cultivation of professional identity. Students described it as the context in which communication skills, professional behaviors, and a sense of "becoming a doctor" were developed, observing that “you're interacting with a patient, and it's probably reflective of a real-life clinical setting… you don't know what to expect, really” (S5) and finding it “very rewarding in terms of the application of your knowledge with an actual patient” (S4). Educators, too, praised bedside teaching for exposing students to real-world complexity and patient unpredictability, as well as cultivating student professionalism, empathy and communication skills:

“There’s nothing like real-life teaching to prepare someone… It’s about courtesy, being polite, but also being focused. You can tell straight away which students have spoken to patients… I’m a huge advocate of bedside teaching.” (E5)

Both groups cited patient availability and time as barriers to effective bedside teaching. Students frequently described large group sizes and unclear participation roles as limiting their opportunities for active involvement: “I was in a group of five… I never had the opportunity to be the person taking the history or doing an examination” (S1). From the educator's perspective, service pressures, time constraints, and space shortages were again highlighted as systemic barriers to delivering regular and high-quality bedside teaching: “Bedside is very helpful for the students, but very hard to organize sometimes. And there's a lot of challenges of patients not being available. It takes a lot of work” (E7). Several educators therefore suggested that neither bedside teaching nor simulation alone was sufficient, arguing instead that a blended model allows each modality to compensate for the other:

“I think both have their advantages. But I don't think one is better than the other. I think you need to have a combination of both.” (E4)

Theme 2: Interactive, case-based, and relational pedagogy best engages students

Students and educators consistently described interactive and relational teaching approaches as more engaging and effective than large lecture-based formats. The latter, whether online or in-person, were described by students as passive and unmemorable; one student described it as “the least effective way of teaching” (S3). Students felt that opportunities for participation, personalization, and feedback were limited, noting that “you can’t necessarily discuss the content that’s being raised” (S6). Support for struggling students was also noted to be limited in lecture-based formats:

“When we're in really big groups, it's often quite difficult then to, if you have a problem, it's quite difficult to express that and get the support that you probably need.” (S3)

Educators largely echoed these concerns, reporting observable disengagement: “It’s hard in a large group… you see people disengaging, disappearing” (E10). They highlighted the limited pedagogical value relative to smaller, interactive sessions where content could be tailored and clinical reasoning nurtured; as one educator explained, “I’ll tend to put them in smaller groups ... so they’re more interactive and more involved” (E1). Case-based in-person teaching was especially praised for its interactivity and relational nature, with one educator remarking, “There’s nothing like real-life teaching to prepare someone” (E5). Conversely, online formats were seen to reduce such opportunities and allow for passivity among students, as described by Educator 4: “the level of feedback, the personal approach…I think that’s what’s lost” (E4).

Student-led and peer teaching also emerged as a valued teaching approach. Students described a reliance on peers and student societies for pastoral support and assessment preparation, reflecting that “student societies (are the) best preparation we’ll get” (S3) and that “if you do need support…you can speak to older peers” (S5). Student 5 emphasized in particular the support offered by peers, explaining that it “wasn’t necessarily the teachers I found support from, more so peers and older colleagues.” Teaching others was also perceived to foster responsibility and deepen understanding, and educators similarly recognized the pedagogical benefit of encouraging autonomy, initiative, and early professional identity formation, with one commenting, “90 percent of my teaching is on the shop floor, and very student-led” (E4). However, both groups stressed the need for educator oversight in order to ensure accuracy and depth of teaching content.

Continuity of teaching staff and the development of an ongoing rapport were also identified as powerful drivers of engagement. Students described greater motivation when educators demonstrated approachability, consistency, and personal recognition, aligning with the view that “sense of belonging makes a difference” (E4). Educators likewise emphasized student-teacher continuity for supporting participation and identifying struggling learners, pointing out that “having a lot of regular contact…helps you to identify those (struggling) students” (E10).

Theme 3: Effective feedback is timely, specific, dialogic, constructive, and actionable

The importance of feedback emerged as a critical theme, with both students and educators identifying its role as central in medical education. Both groups identified that timeliness and specificity were crucial. Feedback during simulation and bedside teaching was particularly praised for its immediacy and potential to stimulate reflection. One educator explained that “they get immediate debrief feedback generated by themselves as part of reflection, along with the debrief facilitators” (E7), emphasizing the value of correcting and reinforcing learning while experiences remain fresh. Delayed or ambiguous feedback was regarded as generic and disconnected from performance, reducing its perceived educational value. As one student expressed, “What was frustrating was I was never given the feedback which explained why” (S1). Another student criticized mock-assessment feedback for being vague, suggesting, “When they won’t tell us what questions we got wrong… that is also not helpful. I need more specifics” (S2).

Participants from both groups highlighted that feedback was most effective when it was delivered as a dialogue rather than a one-directional critique. Students valued being invited to articulate their own reflections before receiving formal feedback, which they felt enhanced ownership of their learning. As Student One explained, “They normally ask, ‘How do you think it went?’ … and then they kind of agree or disagree with what you’ve said.” Educators similarly endorsed this approach, observing that it provided insight into students’ reasoning and reduced defensiveness. One educator also suggested that this approach enhances interactivity, explaining, “I like to have a bit of a back-and-forth kind of dialogue… and try and get them to have discussions among themselves” (E9).

Students and educators each highlighted significant inconsistencies in the availability and quality of feedback. Students particularly cited frustrations that comprehensive feedback was often directed only to struggling students, leaving others without high-quality guidance. One student reflected, “I’m not sure I’ve ever been able to get past the ‘Sorry, we only help people who have failed’” (S1). Educators acknowledged these shortcomings, citing time pressures, competing clinical responsibilities, and limited institutional support as major barriers. As one explained, “We don’t always get the chance to go through things with them” (E2). While personalized written feedback was seen as the "gold standard," educators felt that its delivery was undervalued and unsupported, describing a systemic rather than individual issue rooted in staffing and time constraints.

“I know a lot of my colleagues don't necessarily take part, but that is because of time constraints… There's a whole wealth of knowledge and experience in the senior medical team but because they are busy and job-planned to do so many other things, they can't take more time out to teach students and give thorough feedback.” (E2)

A final issue emerged around the provision of feedback to educators themselves. While educators sought further feedback on their performance, they highlighted that they rarely receive structured evaluation from organizations or learners. One educator voiced concern that “we don’t get any feedback back... That’s a major concern for me in terms of improving medical teaching” (E3). Many described their efforts as invisible and under-recognized, leaving them uncertain about the effectiveness of their teaching, reflected in comments such as, “I’m not even sure I’m doing the right thing” (E3).

Theme 4 - Supporting readiness and motivation requires scaffolding and pastoral care

Students and educators described readiness for clinical learning as highly variable, and educators reported that student confidence in clinical placements appeared lower in the post-pandemic era. Expectations around student autonomy and initiative revealed numerous tensions. Multiple students expressed frustration at a lack of institutional guidance and structure within clinical placements, describing this as detrimental to their learning. One student reported:

“We weren’t given enough of a timetable - we weren’t given a structure. I need structure. When I wasn’t given structure, I panicked and I shut down. And I didn’t do the things that I was supposed to do, because I didn’t know what I was supposed to do. I didn’t know where I was supposed to go.” (S2)

Others reported being left without clear direction on wards, explaining that this left them uncertain about how to contribute or what tasks to undertake: “we’re kind of ignored a lot of the time… when we went to the wards… the junior doctors or the consultants wouldn't have that much time for us” (S3).

Some acknowledged that independence was an expected feature of adult learning, but contended that this adaptive approach is a skill that requires practice over time. Participants described having limited prior experience of autonomous learning, with one student reflecting, “I do genuinely think that is something I found difficult, especially towards the beginning of medical school. I think as you progress through, you learn better how to manage your time” (S6).

Tensions emerged when educators were asked about the same topic: while students looked to educators and institutions for structure and guidance, educators expected students to demonstrate initiative and independence, and expressed clear frustration when learners appeared passive or disengaged. Educator 2 explained that “they want to be just told stuff rather than seek out opportunities”, adding that “they're adults. And they have to make some of their choices themselves”. These differing expectations resulted in both groups viewing the other as disengaged: educators interpreted passivity as a lack of enthusiasm, while students felt that poorly organized teaching reflected educator disinterest, captured in the sentiment, “If (students) feel like the people who are organizing it can't be bothered, then they won't bother themselves” (S1).

Nonetheless, several educators acknowledged the challenges raised by students and endorsed the need for greater support and structure. As one reflected, “They should be guided through. I don't think to give a pass and say, ‘get on with it’ is enough” (E11). Educator 2 recognized the difficulty students face in transitioning to adult learning models, acknowledging that “in school you get told stuff… you get told what to do and then you move to university and you have to do some of that yourself.”

A final dimension of this theme centered on the role of pastoral care in supporting motivation and readiness. Students emphasized its significance for their confidence and well-being, while simultaneously voicing numerous frustrations about the inconsistency of support structures between placements. One participant observed that “the level of support for students varies massively between Trusts…”, later adding “I feel like I can’t access the help I need… there is no consistency” (S2). Students appreciated educators as a critical source of this pastoral support; the same participant went on to state, “I got support not from a system that’s been put in place, but from exceptional individuals who went above and beyond to help me” (S2). Educators affirmed pastoral support as a central part of their role: “it's not all about the education… They need guidance, they need support. They need to feel like they're being looked after, that they are a priority” (E11). They also, however, described this work as an unrecognized emotional labor that added to existing workload pressures. Many noted that their contributions were neither formally acknowledged nor resourced by the organizing institutions, despite being essential to student well-being and engagement. As one educator summarized, “More support on pastoral issues is needed. I think that the depth and the urgency with which issues are dealt with needs to be improved” (E7).

Theme 5: Aligning curriculum and assessment with clinical practice enhances relevance and learning

Curriculum overload was identified by both groups as a substantial barrier to meaningful learning. Students described the volume of content as overwhelming, making effective prioritization and workload management challenging and leaving many feeling lost or "paralyzed." Several attributed this to vague curriculum guidance and the absence of clear standard-setting criteria regarding expected competency levels, noting that, “you're then sort of overwhelmed… and you never get told what you need to know. What is the correct standard for a junior doctor?” (S4). Educators, too, argued that the unrealistic scope of the course encouraged superficial learning, rather than significant depth or understanding of topics, ultimately undermining the students’ ability to apply their knowledge practically. One educator summarized, “the students have a vast curriculum to learn, and I think that can be quite overwhelming” (E4). Student 6 suggested that this has been exacerbated by technology and online resources, describing the cognitive burden of navigating and appraising numerous revision platforms:

“It is very easy to be overwhelmed by the sheer amount of information, and it's difficult to know which information is the best information to be looking at… I think it is very easy to look at all the amount of information out there and feel very overwhelmed and not really sure where to finish.” (S6)

A related concern stemmed from an apparent misalignment between clinical practice and assessment. Students felt that learning on clinical placements did not map directly onto their exam requirements, reducing their motivation to engage: “If this is preparing me to be a foundation year doctor, then I need to be tested on foundation year knowledge” (S2). Educators strongly agreed that assessments emphasized esoteric details at the expense of assessing students’ core practical competencies, observing, “you don't learn to swim in the library…they really need to be out on the shop floor, learning how to do stuff as well as knowing why you do it” (E4). This disconnect was seen as a key driver of disengagement from placements, with students often prioritizing exam revision over authentic clinical learning. One student proposed that “you don’t need practical experience to pass… you could pass with flying colors without seeing a single patient” (S1).

The format and structure of assessments formed an additional source of concern. Despite many students finding reassurance in revision resources that mirror exam blueprints, particularly multiple-choice question (MCQ) banks, both students and educators argued that an overreliance on MCQs encourages superficial, exam-focused learning at the expense of clinical reasoning. As one educator noted, “you can't rely on that alone to get through exams. You need patient interactions to build the full picture of medicine” (E9). Likewise, when reflecting on peers who relied heavily on such revision strategies, one student recognized that “their understanding is very fragile. So when they're probed on it in the OSCE or in bedside teaching or by a consultant, it falls apart” (S4). Several participants suggested short-answer questions, problem-based, or viva-style assessments as alternatives or adjuncts to MCQs, and praised well-executed objective structured clinical examinations (OSCEs) as “a really great way to examine your competencies” (S1).

## Discussion

Our five overarching themes align with existing literature on medical teaching and learning and highlight practice-relevant considerations arising from this evaluation.

Integrating online, simulation, and bedside modalities for coherent learning

Our findings align with existing evidence supporting the utility of a hybrid approach that integrates both online and in-person modalities. In a large national survey, Dost et al. reported that students favored a blended model combining both face-to-face and online teaching components [5]. Similarly, Zhao et al. observed that hybrid programs achieved the highest student satisfaction ratings, whereas exclusively online or face-to-face models were associated with markedly lower satisfaction [14]. Taken together with this wider evidence, these accounts suggest that online lectures and seminars may be retained for their accessibility and flexibility, but that they be redesigned to address features associated with reduced engagement in our data, such as limited interactivity, reduced opportunities for real-time feedback, and the ease with which students can remain passive in virtual settings. For example, sessions might be delivered live and be recorded for later review, or features such as polling and guided discussion could be embedded to promote active engagement [15]. According to our own findings, fully pre-recorded online sessions added little value amid the abundance of existing online resources and therefore may be unsuited to replacing interactive formats in contemporary curriculum design.

The complementary use of different clinical teaching modalities emerged as an approach of particular value. While students’ appraisals defined bedside teaching and simulation as separate entities, educators concluded that many limitations of either approach are complemented by the strengths of the other. Previous literature is limited in its focus on the importance of this blended approach to clinical teaching. Elendu et al. highlight the potential benefits of integrating simulation with other modalities, but comments are limited to speculation only [16]. This evaluation adds a contextual qualitative perspective by highlighting the potential value of a sequential blend of modalities, in which early simulation develops a foundation of competence and confidence that may facilitate a smoother transition into authentic bedside learning. Furthermore, our findings support the pedagogical value of bedside teaching, which is particularly salient given reports of its declining incorporation into medical curricula [2,17]. In this way, these findings support the value of safeguarding and integrating bedside teaching within structured, blended curricula.

Prioritizing interactive and case-based approaches to strengthen engagement

Students showed a strong inclination towards interactive, case-based small-group sessions over didactic lectures, which they perceived as passive and unengaging despite their organizational convenience. This preference for interactive and case-based small-group teaching aligns with existing literature, which has demonstrated how active learning strategies enhance performance and encourage deeper understanding and collaboration [18].

The interviewees also emphasized the value of their peers as sources of emotional and academic support, with peer-assisted learning shown to improve exam performance [19]. Further qualitative data, alongside the findings of this evaluation, describe the pastoral benefits of peer-assisted learning in improving confidence and readiness to integrate with the clinical environment [20]. Collectively, these findings suggest value in promoting peer-based learning through formal mentoring schemes or structured student-led study groups, with appropriate oversight and training for senior students to support equitable access and consistency of teaching.

Embedding structured feedback processes to support learner progress

The provision of feedback emerged as a highly valued aspect of the educational process for both students and educators. This was valued by students when it was specific, immediate, and framed in a two-way conversation; the absence of these characteristics, particularly following assessments, caused significant demotivation. These findings are supported by published literature suggesting that effective feedback should be actionable, constructive, and viewed as an ongoing conversation between educator and learner [21]. Training staff to deliver feedback in this way may help encourage an environment that facilitates discussion and builds trust between educators and students. These accounts support both the consideration of protected time for debriefing within teaching schedules, and the development of clear, standardized expectations to enhance feedback quality and promote consistency across placements.

In this evaluation, educators reported that they received minimal feedback from students and limited recognition of their work from their institutions, which they felt constrained their own professional development. A potential explanation for this lack of educator feedback is offered by Wisener et al., who conducted focus groups with medical students [22]. Their findings suggest that a perceived imbalance of power and concerns about a lack of anonymity when providing feedback results in reluctance among medical students to provide feedback to their educators [22]. While these barriers to educator-focused feedback are not easily addressed through any single intervention, integrating anonymous and user-friendly means of delivering feedback, such as electronic forms, may help foster honest and constructive student responses, which may better support educator development.

Providing scaffolding and support to improve confidence and clinical readiness

The successful delivery and reception of education has been shown to be influenced by readiness and motivation [23], a sentiment strongly echoed by participants in this evaluation. Student interviewees described uncertainty and reduced confidence during patient interactions, consistent with post-pandemic reports of reduced self-efficacy [24]. Although some acknowledged the autonomy afforded by clinical placements, others felt that the transition from structured preclinical teaching to largely self-directed workplace learning lacked sufficient scaffolding, leaving students feeling disoriented, overwhelmed, and unprepared. These accounts indicate that initiative should not be regarded as an "innate" attribute, but rather as a learned skill that requires practice, cultivation, and support. Students and educators alike called for clearer timetables, defined roles, and explicit induction on how to optimize their learning in clinical environments. Our findings point to these as examples of early, structured support, which may provide a platform onto which students can build the confidence and autonomy that enables them to engage more actively in clinical learning.

The accounts of both students and educators highlight pastoral support as an important determinant of readiness for clinical learning. However, the students described substantial inconsistency in the availability and quality of support between placements. Our data suggest that, in this context, greater consistency in support might be promoted through a clear, centralized point of contact for wellbeing support, with transparent referral pathways and consistent visibility of available services. Such approaches may help address students’ concerns that the issue lies not in the absence of support, but in its poor accessibility. As previously discussed, educators described the burden of providing pastoral support without institutional recognition of this additional role. Protected time and training in relation to this role may help ensure that educators and students have a safe and supportive working environment.

Strengthening curriculum-assessment alignment to promote practice-relevant learning

Our findings suggest that curriculum overload remains a significant perceived barrier to meaningful learning, with vague guidance and unclear competency standards leaving students uncertain about the level of knowledge expected of them. This barrier to learning is compounded by a perceived misalignment between clinical learning and medical school assessment, where students feel that examinations emphasize esoteric details rather than the core competencies required of a junior doctor, thereby reducing their motivation to engage with authentic clinical experiences. Biggs’ theory of constructive alignment provides a useful lens through which to interpret these findings, asserting that learning outcomes, teaching methods, and assessments must be coherently integrated to sustain motivation and guide student effort [25]. Educators expressed frustration with students’ exam-focused behavior. However, these findings raise the possibility that assessment-driven behavior could be more constructively harnessed by the explicit blueprinting of assessments to practice-relevant competencies such as clinical reasoning, communication, and professionalism. Closer alignment between assessments and competencies attained through clinical practice may help encourage student engagement with clinical placements. This interpretation is supported by the work of Anderson et al., who demonstrated that alignment between objectives, active learning, and assessment can effectively promote student engagement [26]. These findings also suggest that streamlining curricula may help reduce student overload, aligning with evidence that practical curriculum mapping can identify redundancies and improve curriculum coherence [27].

The participants from each group criticized an overreliance on MCQs during examinations, which were felt to encourage superficial learning at the expense of clinical reasoning and communication skills. While MCQs are useful for assessing factual knowledge and breadth of understanding, a study by Khan et al. highlighted significant misalignment between the content covered by MCQs and curriculum objectives [28]. Complementary formats such as short-answer questions, problem-based assessments, or structured viva examinations, all of which were suggested as alternatives by participants, were perceived to better assess reasoning, communication, and professionalism while retaining reliability and objectivity. These findings may have relevance to the evolving UK Medical Licensing Assessment (UKMLA) curriculum [29], as both groups of participants called for clearer specification of the level of competence and depth of knowledge expected within the UKMLA content map. Preston et al. observed that without these transparent assessment expectations, examinations risk driving superficial study [30]. Greater clarity regarding the scope and complexity of MCQ examinations may support students in prioritizing clinical reasoning, communication, and professionalism, rather than focusing disproportionately on breadth of factual recall.

Limitations

As this was a local service evaluation based on a purposive, non-random sample of students and educators from a limited number of UK medical schools and affiliated institutions, the findings should be interpreted as exploratory and hypothesis-generating. Although this evaluation offers insights relevant to undergraduate clinical education settings, the findings may not be transferable to all medical teaching contexts. The small number of student participants may have limited the range of experiences and attitudes captured, and the voluntary basis of participation might have resulted in a sample consisting of students who were more engaged or strongly opinionated than their peers, which may have influenced the range and balance of views represented. In addition, participant characteristics that may shape educational experiences, such as neurodivergence, were not systematically collected, and this may have limited the study’s ability to explore differences in how teaching and support were experienced across learner groups. A further limitation relates to the use of AI-assisted preliminary coding. Although all AI-generated outputs were manually reviewed against the original transcripts and final analytical decisions were human-led, AI tools may be less sensitive to contextual nuance and latent meaning than human researchers. As a result, some subtleties within participants’ accounts may have been under-recognized during early coding. This risk was mitigated through iterative human verification, reflexive engagement, and human-led development of the final codebooks, subthemes, and themes.

Future research

Given the scope of this evaluation, further qualitative studies of specific student groups whose experiences of medical education may be systematically different, such as neurodiverse students or students from minority backgrounds, are warranted. Further study is also needed to explore the optimal sequencing of blended learning modalities (in particular, those combining simulation and bedside teaching), and to examine how the continuity of teaching staff and institutional support influences student engagement and wellbeing. Quantitative investigations may also be of value, to determine whether an improved alignment between assessment, teaching, and practice objectively enhances readiness for clinical and professional practice.

## Conclusions

Students and educators described several aspects of undergraduate clinical education that may be misaligned with their perceived values and priorities. Both students and educators described preferences for a structured, practice-aligned model that integrates online and in-person modalities and the sequential use of simulation and bedside learning, underpinned by timely feedback, guided reflection, and relational, case-based teaching. Readiness for clinical learning was described as being shaped by scaffolded autonomy, clear structure, and meaningful pastoral support, complemented by accessible peer networks. Participants identified misalignment between their curriculum, clinical placements, and assessments as a key barrier to meaningful engagement and preparation for practice. Closer alignment of teaching and assessment with clinical competencies may help address this divide in similar medical education settings, but this requires further study.

## Appendices

Appendix A. Semi-structured interview guide for medical educators

To be conducted online via Teams/Zoom, with the option of an in-person meeting if the participant has poor Internet access.

Introduction

□ Thank the participant for their time.

□ Explain the purpose of the interview - to explore their experiences and insights related to teaching medical students.

□ Explain that the interview contains 21 questions and should take less than an hour to complete.

□ Assure participants that participation is voluntary, that they may stop the interview or withdraw at any time, and that their responses will be de-identified, kept confidential, and securely stored in accordance with institutional policy.

□ Obtain verbal informed consent for audio recording, transcription, analysis, and the use of de-identified anonymized quotations in analysis and publication.

Section 1: Background information

1. Can you tell me about your role in teaching medical students?

2. What type of medical students do you typically teach? (If the participant requires further prompting: e.g., preclinical, clinical, postgraduate).

Section 2: Teaching methods

1. How would you describe your overall teaching approach with medical students?

2. What specific methods or techniques do you find most effective in teaching medical students?

3. How do you assess student learning and performance in your courses?

Section 2.5: Bedside vs simulation-based teaching - if the educator has not provided a sufficiently detailed answer in Section 2

1. What are your thoughts on bedside teaching and simulation-based teaching, and which do you prefer delivering?

a. In your opinion, what are the pros and cons of each?

b. What are the challenges you face in delivering each of these teaching sessions?

c. Are there any methods you use to overcome these challenges? 

Section 3: Student engagement and challenges

1. In your experience, what are the common challenges medical students face in the learning process?

2. How do you motivate students to engage with the material, particularly in large group settings?

3. Can you share any strategies you use to support struggling students or address learning difficulties?

Section 4: Technology and innovation

1. How do you use technology in your teaching? (If the participant requires further prompting: e.g. online learning platforms, simulation tools, or digital resources).

2. In what ways do you think technology has enhanced or hindered medical education?

Section 5: Feedback and improvement

1. How do you provide feedback to students on their performance?

2. What kinds of feedback do students typically find most helpful?

3. Have you received feedback from your students on your teaching methods? If so, what have you learned from it?

Section 6: Challenges and opportunities

1. What (other) challenges or barriers do you face in delivering your teaching sessions, and how do you overcome them? (Prompts: lectures/seminars, bedside, simulations if relevant).

2. Are there any methods you use to overcome these challenges?

3. Are there any resources or support that you wish was available to help improve your experience of teaching?

Section 7: Closing

1. What do you find most rewarding about teaching medical students?

2. What advice would you offer to other educators in medical school settings?

3. What improvements or changes would you like to see in medical education, either in terms of curriculum, teaching methods, or institutional support?

4. Is there anything you would like to add on the topic of medical teaching that we have not covered in the interview?

Appendix B. Semi-structured interview guide for medical students

To be conducted online via Teams/Zoom, with the option of an in-person meeting if the participant has poor internet access.

Introduction

□ Thank the participant for their time.

□ Explain the purpose of the interview - to explore their experiences and insights related to medical education.

□ Explain that the interview contains 20 questions and should take less than an hour to complete.

□ Assure participants that participation is voluntary, that they may stop the interview or withdraw at any time, and that their responses will be de-identified, kept confidential, and securely stored in accordance with institutional policy.

□ Obtain verbal informed consent for audio recording, transcription, analysis, and the use of de-identified anonymised quotations in analysis and publication.

Background information

Background information can be found on the participant’s survey response. Additional questions:

1. What is your preferred learning style? (If the participant requires further prompting: e.g. visual, auditory, or kinaesthetic).

Section 1: Free association task

Please give the first four words or expressions that come to your mind when you hear the following phrases:

1. Online learning

2. Simulation teaching

3. Bedside teaching

Section 2: Experience with teaching

1. How do you feel about the teaching methods used in your medical school? (If the participant requires further prompting, available prompts include: “How do you feel about…”)

□ Lectures (both online and in person)

□ Seminars (both online and in person)

□ Simulation teaching

□ Bedside teaching

2. Can you describe a teaching method or experience that you found particularly effective or engaging?

3. Conversely, have there been teaching methods or approaches that you found less effective or engaging?

Section 2.5: Bedside vs simulation-based teaching - if the student has not provided a sufficiently detailed answer in Section 2

1. What are your thoughts on bedside teaching and simulation-based teaching, and which do you prefer?

a. In your opinion, what are the pros and cons of each?

b. What are the challenges you face learning during each of these teaching sessions?

c. How do you think these challenges could be overcome?

d. Are there any methods you use to overcome these challenges? 

Section 3: Student engagement and learning

1. How do you feel about the balance between theoretical knowledge and practical/clinical skills in your education?

2. In what ways do you feel supported (or unsupported) by your teachers in your learning process?

Section 4: Feedback and assessment

1. How is feedback provided to you about your performance?

a. Is it helpful?

b. Why or why not?

2. How do you typically prepare for exams or assessments in medical school?

a. Are there ways in which your medical educators could better prepare you for these exams?

3. Do you feel that the assessments (e.g., written exams, OSCEs, clinical evaluations, essays) reflect your learning and understanding effectively?

a. How could these be adjusted to better reflect your knowledge, understanding, and abilities?

Section 5: Use of technology

1. How do you use technology in your studies? (If the participant requires further prompting: e.g. online resources, virtual patient cases, or learning apps).

2. Would you say technology has helped or hindered your learning?

Section 6: Challenges and opportunities

1. What challenges or barriers do you face in your medical education, and how do you overcome them?

2. Are there any resources or support systems that you wish were available to help improve your learning experience?

Section 7: Closing

1. What do you find most rewarding about your medical education?

2. If you could change anything about your medical school’s teaching approach, what would it be?

3. Is there anything you would like to add on the topic of medical teaching that we have not covered in the interview?
